# Comparative efficacy and safety of vonoprazan-based dual therapy vs. bismuth-based quadruple therapy for *Helicobacter pylori* eradication: a GRADE-assessed systematic review and meta-analysis with trial sequential analysis

**DOI:** 10.3389/fmicb.2026.1872669

**Published:** 2026-08-24

**Authors:** Alejandro Chen Liang, Yeison Cruz Castillo, Lorena M. Murrieta Bruciaga, Barbara Abreu Lopez, Salma Beltrán Covarrubias, Hector Jonan Flores Uribe, Vanessa Pamela Salolin-Vargas, Gabriela Flores Monar, José García-Corella, Ishaan Kalha, Ernesto Calderon Martinez

**Affiliations:** 1 Centro de Estudios Universitarios Xochicalco, Campus Tijuana, Tijuana, México; 2 Universidad Autónoma de Santo Domingo, Santo Domingo, República Dominicana; 3 Universidad del Valle de México, Hermosillo, México; 4 Maimonides Medical Center, New York, NY, United States; 5 Universidad de Guadalajara, Guadalajara, México; 6 Facultad de Medicina, Universidad Westhill, Ciudad de México, México; 7 University of Miami/Jackson Health System, Miami, FL, United States; 8 Department of Medicine, UCLA-Kern Medical, Bakersfield, CA y David Geffen School of Medicine at UCLA, Los Angeles, CA, United States; 9 Division of Gastroenterology, Department of Medicine, UCLA-Kern Medical, Bakersfield, CA y David Geffen School of Medicine at UCLA, Los Angeles, CA, United States; 10 Texas Tech University Health Sciences Center El Paso, El Paso, TX, United States

**Keywords:** adverse events, bismuth quadruple therapy, compliance, eradication rate, vonoprazan–amoxicillin dual therapy

## Abstract

**Background:**

*Helicobacter pylori* infects approximately 4. 4 billion individuals worldwide, causing chronic gastritis, peptic ulcer disease, and gastric adenocarcinoma. The 2024 ACG guideline recommends bismuth-based quadruple therapy (BQT) as first-line treatment when susceptibility is unknown, while vonoprazan–amoxicillin (VA) dual therapy is conditionally endorsed as an alternative. Prior meta-analyses included vonoprazan-containing BQT control arms, introducing heterogeneity that may influence comparative efficacy estimates. This analysis addresses that gap by restricting the comparator to conventional PPI-based BQT.

**Methods:**

A systematic search of PubMed/MEDLINE, EMBASE, Web of Science, CINAHL, Google Scholar, and the Cochrane Library was conducted through April 2026 (PROSPERO: CRD420251108849) for randomized controlled trials (RCTs). All comparator arms were restricted to conventional PPI-based BQT. Outcomes included eradication rate, total adverse events (AE), nausea, diarrhea, treatment compliance, and bitter taste. Certainty of evidence was assessed using the GRADE approach and trial sequential analysis. Meta-analysis was performed using R software.

**Results:**

From 17,661 screened articles, 15 RCTs comprising 4,410 patients were included. Pooled eradication rates were 86.4% with VA and 85.0% with BQT, with no statistically significant difference (RR 1.03, 95% CI 1.00–1.07; *p* = 0.05; *I*^2^ = 46.1%). VA significantly reduced total AEs (17.1% vs. 35.4%; RR 0.48, 95% CI 0.42–0.54; *p* < 0.01; *I*^2^ = 0.0%), nausea (4.4% vs. 10.6%; RR 0.44, 95% CI 0.32–0.59; *p* < 0.01; *I*^2^ = 26.7%), and bitter taste (5.6% vs. 14.5%; RR 0.10, 95% CI 0.06–0.18; *p* < 0.01; *I*^2^ = 43.2%). No significant differences were observed for diarrhea or compliance. GRADE certainty was moderate for eradication, low for AEs, nausea, and bitter taste, and very low to low for diarrhea and compliance. Trial sequential analysis confirmed sufficient evidence for reductions in AEs, nausea, and bitter taste, while eradication, diarrhea, and compliance remained inconclusive.

**Conclusion:**

VA dual therapy achieves eradication rates comparable to PPI-based BQT while offering a significantly more favorable tolerability profile, driven by fewer total AEs, less nausea, and less bitter taste. As all included trials were conducted in China, generalizability requires further investigation. These findings support VA dual therapy as a viable alternative for first-line *H. pylori* management.

**Systematic review registration:** https://www.crd.york.ac.uk/prospero/display_record.php?RecordID=1108849, identifier: CRD420251108849.

## 1 Introduction

*Helicobacter pylori*, colonizes the human stomach and remains a major pathogen in gastroenterology and infectious diseases (Bashir and Khan, 2023; Ali and AlHussaini, 2024). It is the most common bacterial infection occurring in ~4.4 billion individuals (~50% of the global population; Bashir and Khan, 2023). Prevalence varies by geography, socioeconomic status, hygiene, and environmental factors, with oral transmission as the primary route (Ali and AlHussaini, 2024). Rising antibiotic resistance, exacerbated by inappropriate prescriptions, has increased the global burden (Bashir and Khan, 2023). Its pathogenesis drives chronic gastric inflammation, peptic ulceration, and progression to gastric adenocarcinoma (Ali and AlHussaini, 2024). According to the 2024 American College of Gastroenterology (ACG) guidelines, bismuth-based quadruple therapy (BQT) is the first-line regimen. Alternatives include rifabutin-based triple therapy, and vonoprazan-amoxicillin (VA; Chey et al., 2024). BQT achieves eradication rates approaching 90%, though its four-drug administration schedule, gastrointestinal adverse event (AE) burden, and cost represent meaningful barriers to adherence (Aldhaleei et al., 2024). Vonoprazan (VPZ), a potassium-competitive acid blocker (PCABs), provides rapid, sustained inhibition of gastric H^+^/K^+^-ATPase, maintaining gastric pH to 6–7, enhancing antibiotics efficacy such as amoxicillin (AMX), clarithromycin, and tetracycline (Chey et al., 2024; Shirley, 2024; Chey et al., 2022). Unlike PPIs, VPZ is not subject to CYP2C19 polymorphisms, is food-independent, and demonstrates consistent pharmacokinetics across patient populations (Shirley, 2024). In the context of rising clarithromycin resistance, PPI–clarithromycin triple therapy has lost effectiveness, whereas VPZ-based therapies provide more reliable outcomes (Shirley, 2024). This analysis focuses on VA dual therapy, supported by moderate-strength evidence in the 2024 ACG guideline (Chey et al., 2024). Prior meta-analyses on this comparison, including (Li et al. 2025) and (Zhou et al. 2024) incorporated RCTs in which VPZ, rather than a conventional PPI, served as the acid suppressant within the BQT control arm (Chen Liang et al., 2025). Given that VPZ-based BQT provides pharmacologically superior acid suppression relative to PPI-based BQT, its inclusion as a comparator attenuates the apparent benefit of VA dual therapy and introduces systematic heterogeneity that compromises pooled estimates. To address this limitation, the present analysis restricted the comparator exclusively to conventional PPI-based BQT. Additionally, VA dual therapy offers simplified dosing, reduced pill burden, and no requirement for clarithromycin susceptibility testing, positioning it as a practical first-line option in settings with high clarithromycin resistance or limited adherence. We further incorporated GRADE certainty-of-evidence grading and trial sequential analysis (TSA) to determine whether current evidence is sufficient to draw definitive conclusions or whether additional trials remain necessary.

## 2 Methodology

The present systematic review followed the recommendations and criteria established by the Preferred Reporting Items for Systematic Reviews and Meta-analyses (PRISMA) reporting guidelines (Page et al., 2021). We pre-registered the protocol at the International Prospective Register of Systematic Review (PROSPERO) with the identifier code CRD420251108849 (NIHR, n.d.). Clinical trial number: not applicable.

### 2.1 Eligibility criteria

This review included studies published from the inception of the database to April 2026, restricted to those available in English. Eligible study designs included only RCTs. Additionally, studies were excluded if they lack sufficient methodological detail, present duplicate data, or fail to provide the required information after attempts to contact the original authors. The study population included adults with *Helicobacter pylori* infection. Eligible studies compared vonoprazan-amoxicillin (VA) dual therapy, administered at any dose, with bismuth and BQT. The assessed outcomes included the efficacy outcome, eradication rate (ER), and safety outcomes, total AEs considering nausea/vomiting, diarrhea, bitter taste, and treatment compliance.

### 2.2 Searching methods

We conducted a systematic search on April 2026, on PubMed/MEDLINE, EMBASE, Web of Science, Google Scholar, CINAHL and Cochrane using the following terms: “*Helicobacter pylori*,” “Vonoprazan,” “Amoxicillin,” “Bismuth Quadruple Therapy,” “Eradication Rate,” among others. We have provided all detailed search strategies in Supplementary Tables 1–6.

### 2.3 Selection of studies

We exported all references to Rayyan (Rayyan Systems Inc., Cambridge, MA, USA) and removed duplicates (Rayyan, n.d.). Two authors independently (V.P.S.V and L.M.M.) completed the eligibility assessment, first by title and abstract analysis and, subsequently, by full-text assessment. In disagreements between reviewers, a third reviewer (Y.C.C.) helped reach a consensus.

### 2.4 Data extraction

Two independent reviewers extracted the data (L.M.M. and Y.C.C.). When multiple reports from the same study existed, we included the most complete report. Extracted data included sample sizes, intervention types, and measured outcomes. We employed conventional data extraction methods, complemented by specialized tools such as WebPlotDigitizer (Automeris, Austin, TX, USA) for digitizing data from graphs, Cochrane Calculator for statistical conversions, and StatsToDo for advanced calculations (automeris.io, n.d.; RevMan Calculator | Cochrane Training, n.d.; StatsToDo, n.d.). The assessed outcomes included eradication rate (ER), total AEs, nausea, diarrhea, and treatment compliance. Additional extracted data included subgroup characteristics, such as country of study, risk of bias levels, and study design.

### 2.5 Assessment of risk of bias in included studies

To assess the quality of the studies included in the systematic review, according to the Cochrane guidelines, we applied the Cochrane RoB 2.0 tool for RCTs (Risk of Bias Tools, n.d.). Two independent reviewers (L.M.M. and Y.C.C.) evaluated the risk of bias in each study, considering the specific criteria and guidelines provided by the respective tools. We resolved discrepancies between reviewers through discussion with a third, blinded reviewer (S.A.B.).

### 2.6 Assessment of the certainty of the evidence and summary of findings

Two reviewers (S.A.B. and A.C.L.) independently assessed the overall quality of evidence for each outcome using the five domains of the Grading of Recommendations Assessment, Development, and Evaluation (GRADE) approach study design, risk of bias, inconsistency, indirectness, imprecision, and publication bias, large effect, plausible confounding, dose response gradient, via the GRADEpro GDT software (GRADEpro, n.d.; Schünemann et al., n.d.). In disagreements between reviewers, a third reviewer (Y.C.C.) helped reach a consensus.

### 2.7 Statistical analysis

Meta-analyses were performed using R software version 4.3.3 (R Foundation for Statistical Computing, Vienna, Austria) with the *meta, metafor*, and *dmetar* packages (RStudio Desktop, n.d.; R, n.d.). Dichotomous outcomes were pooled using random-effects meta-analysis through the inverse variance method implemented in the metabin() function. Effect estimates were expressed as relative risks (RRs) with 95% confidence intervals (CIs). Whenever an insufficient number of studies reported an outcome of interest, only a qualitative synthesis was performed. Quantitative analyses were conducted according to outcome-specific data availability. Heterogeneity was assessed using the *I*^2^ statistic, with values of < 25%, 25–50%, and >50% representing low, moderate, and substantial heterogeneity, respectively. Publication bias was evaluated through funnel plot inspection and Egger's regression test when ≥10 studies were available. Subgroup analyses were conducted according to amoxicillin dosage, amoxicillin frequency, comparator-based regimens, clarithromycin-based comparator regimens, treatment duration, alcohol and smoking status, and risk of bias, when applicable. Sensitivity analyses were performed using leave-one-out influence analysis and additional exclusion analyses when appropriate. Absolute event rates were additionally summarized descriptively using pooled crude event counts across studies. Additionally, Trial Sequential Analysis (TSA) was conducted using TSA software version 0.9.5.10 Beta (Copenhagen Trial Unit, Copenhagen, Denmark) to assess the conclusiveness of pooled evidence and determine whether additional studies were required (Thorlund et al., 2011). Required information size (RIS) was estimated based on anticipated intervention effects, control event rates, and diversity-adjusted heterogeneity. TSA monitoring boundaries were used to determine whether evidence was conclusive or whether further studies were needed.

## 3 Results

### 3.1 Study selection

**Figure 1:**
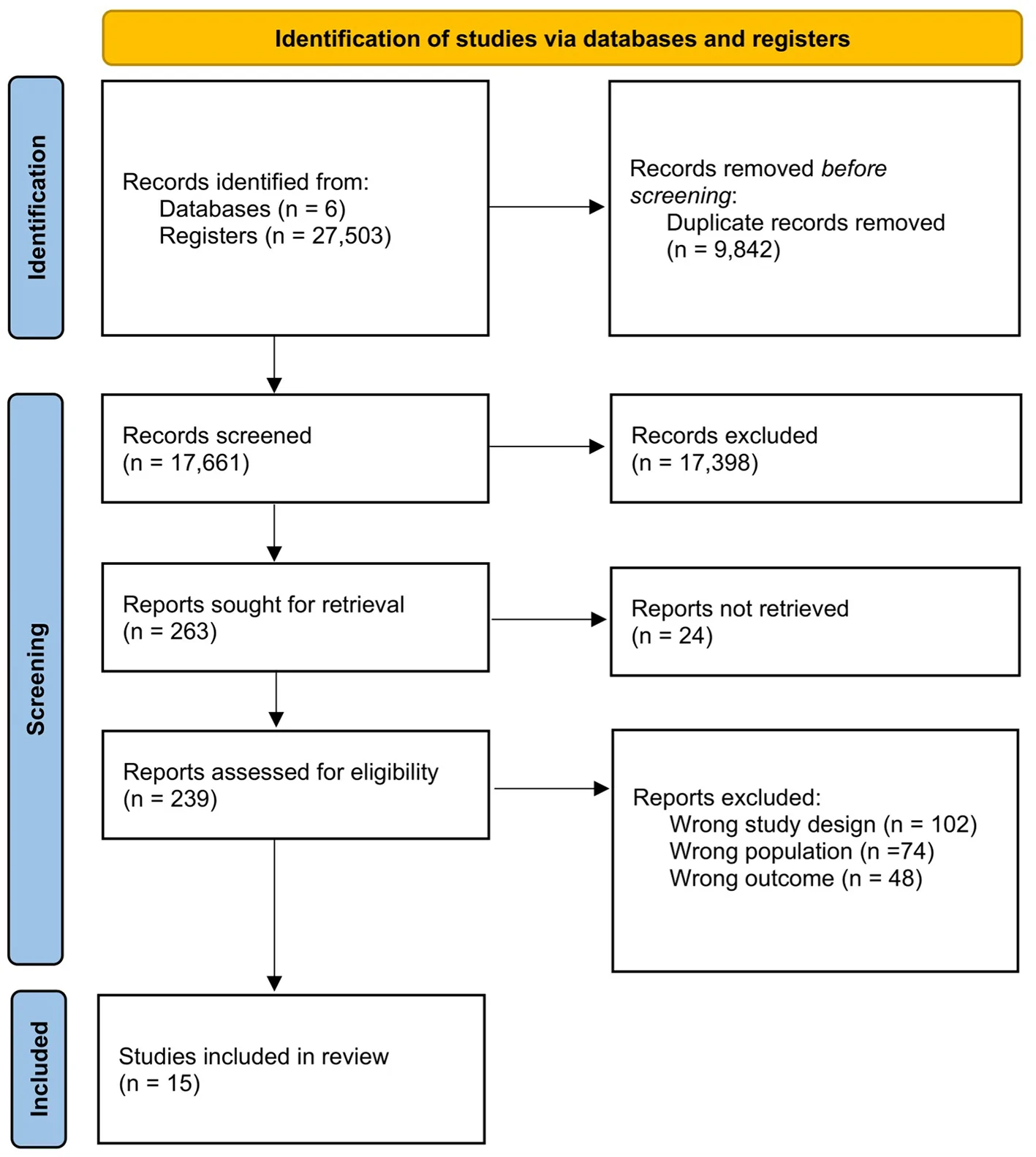
PRISMA flow diagram: 27,503 records identified from six databases/registers, 17,661 records screened, and 15 studies included in the review.

In our initial search, we identified 27,503 records across six databases and registers. After removing 9,842 duplicate records, 17,661 records remained for title and abstract screening. We then sought 263 reports for retrieval; however, 24 reports could not be retrieved, leaving 239 reports for eligibility assessment. These full-text reports underwent eligibility screening, excluding 102 due to wrong study design, 74 due to wrong population, and 48 due to wrong outcome. Ultimately, 15 studies were included in this review. We summarized this process in our PRISMA flow diagram (Figure 1).

### 3.2 Characteristics of included studies

We analyzed data from 15 studies involving 4,410 patients: 2,204 received VA dual therapy and 2,206 received the BQT standard therapy. All studies were conducted in China, reflecting a geographically homogenous study population. For eradication rates, 33% of studies favored VA dual therapy, while 67% reported no difference compared with BQT therapy. Safety outcomes consistently favored VA dual therapy, where 93% of studies reported fewer overall AE, with only 7% reporting no difference. Among individual AE, 67% of studies favored VA dual therapy for lower nausea incidence, 26% reported no difference, and 7% did not assess this outcome. For diarrhea, 33% favored VA dual therapy, 53% found no difference, and 14% did not report this endpoint. Compliance was less frequently evaluated; 7% of studies favored VA dual therapy, 60% of studies found no difference; 33% did not report data, and none favored either regimen. Bitter taste was also infrequently evaluated, with 40% of studies favoring VA dual therapy, 13% reporting no difference, and 47% not reporting this outcome; again, none favored either regimen. We summarized this information in Table 1 (Chen et al., 2024; Cheng et al., 2026; Cheung et al., 2024; Fan et al., 2025; Hu et al., 2023; Huang et al., 2024; Jiang et al., 2024; Li et al., 2023; Peng et al., 2023; Qian et al., 2023; Wang et al., 2023; Yan et al., 2024; Yang et al., 2023; Zhang et al., 2025a,b).

**Table 1:** General outcomes of included studies comparing vonoprazan-amoxicillin dual therapy vs. bismuth-based quadruple therapy.

Author and year	Country	Study design	Intervention	Comparator	Follow up (weeks)	Total sample size	Treatment duration (days)	Eradication rate (%)	

								VPZ	BQT
(Chen et al. 2024)	China	RCT	VPZ 20 mg (BID) + AMX 1.0 g (TID)	BPC 240 mg + Ilaprazole 5 mg + AMX 1.0 g + FZD 100 mg (BID)	4	90	14	84.4	84.4
(Cheng et al. 2026)	China	RCT	VPZ 20 mg (BID) + AMX 1.0 g (TID)	BPC 600 mg + Esomeprazole 20 mg (BID) + AMX 1 g + Clarithromycin 500 mg (BID)	4	300	10	88	85.3
(Cheung et al. 2024)	China	RCT	VPZ 20 mg (BID) + AMX 1.0 g (TID)	BPC 220 mg + Esomeprazole 20 mg (BID) + TTC 500 mg (TID) + Metronidazole 500 mg (QID)	6	200	14	96	92
(Fan et al. 2025)	China	RCT	VPZ 20 mg (BID) + AMX 1.0 g (BID)	BPC 220 mg + Esomeprazole 20 mg (BID) + AMX 1 g + Clarithromycin 500 mg (BID)	4	504	14	79.4	85.7
(Hu et al. 2023)	China	RCT	VPZ 20 mg (BID) + AMX 1.0 g (TID)	BPC 220 mg + Esomeprazole 20 mg + AMX 1 g (BID) + Metronidazole 0.4 g (QID)	6	194	14	88.6	91.7
(Huang et al. 2024)	China	RCT	VPZ 20 mg (QD) + AMX 1.0 g (TID)	BPC 220 mg + Ilaprazole 10 mg + AMX 1 g + Clarithromycin 500 mg (BID)	6	203	14	92.2	80.2
(Jiang et al. 2024)	China	RCT	VPZ 20 mg (BID) + AMX 1.0 g (TID)	BPC 0.6 g + Rabeprazole 10 mg + AMX 1 g + Clarithromycin 500 mg (BID)	4	400	14	94	87
(Li et al. 2023)	China	RCT	VPZ 20 mg (BID) + AMX 750 mg (TID)	BPC 240 mg + Esomeprazole 20 mg + AMX 1.0 g + FZD 100 mg (BID)	4	150	14	77.3	78.6
(Peng et al. 2023)	China	RCT	VPZ 20 mg (BID) + AMX 750 mg (QID)	BPC 220 mg + Esomeprazole 20 mg + AMX 1 g + Clarithromycin 500 mg (BID)	4	316	14	89.9	81
(Qian et al. 2023)	China	RCT	VPZ 20 mg (BID) + AMX 750 mg (QID)	BPC 200 mg + Esomeprazole 20 mg + AMX 1 g + Clarithromycin 500 mg (BID)	6	250	10	82.4	88
(Wang et al. 2023)	China	RCT	VPZ 20 mg (BID) + AMX 750 mg (QID)	BPC 220 mg + Rabeprazole 10 mg + AMX 1 g + Clarithromycin 500 mg (BID)	6	151	14	94.6	87
(Yan et al. 2024)	China	RCT	VPZ 20 mg (BID) + AMX 1.0 g (TID)	BPC 200 mg + Rabeprazole 10 mg + AMX 1 g + Clarithromycin 500 mg (BID)	12	314	10	86	89.2
(Yang et al. 2023)	China	RCT	VPZ 20 mg (BID) + AMX 1.0 g (TID)	Rabeprazole (20 mg) bid + BPC/tinidazole/Clarithromycin Combined package 4.2 g (BID)	6	400	14	92.5	81.5
(Zhang et al. 2025a)	China	RCT	VPZ 20 mg (BID) + AMX 1.0 g (TID)	BPC 220 mg + Esomeprazole 40 mg + FZD 100 mg (BID) + TTC 500 mg (TID)	NA	688	14	73.8	76.2
(Zhang et al. 2025b)	China	RCT	VPZ 20 mg (BID) + AMX 1.0 g (TID)	BPC 240 mg + Lasoprazole 30 mg + AMX 1 g + Clarithromycin 500 mg (BID)	4	250	14	92	88

AMX, amoxicillin; BID, twice daily (from Latin bis in die); BPC, bismuth potassium citrate; BQT, bismuth quadruple therapy; QID, four times daily; VA, vonoprazan–amoxicillin dual therapy regimen; VPZ, vonoprazan.

### 3.3 Risk of bias

**Figure 2:**
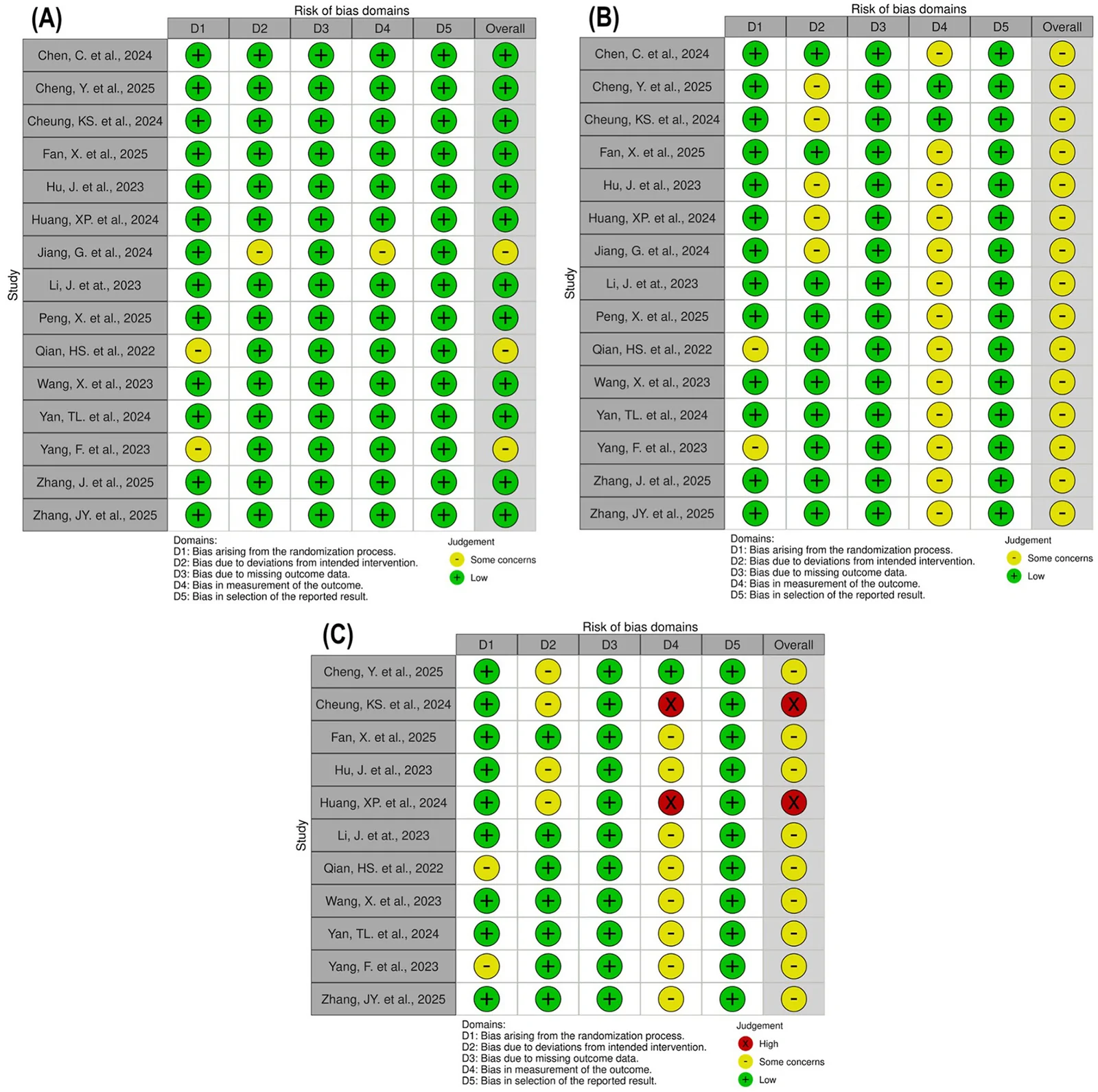
Risk of bias assessment of the outcomes: **(A)** eradication rate, **(B)** adverse events (total adverse events, nausea, diarrhea and bitter taste), **(C)** treatment compliance.

Risk of bias was assessed using the RoB 2 tool (Risk of Bias Tools, n.d.) on an outcome-specific basis across the 15 included randomized controlled trials. For eradication rates, reported by all 15 studies, 12 were judged at low risk of bias (80.0%), while three (20.0%) were rated as having some concerns (Figure 2A). For adverse events (including total adverse events, nausea, diarrhea, and bitter taste), reported by 15 studies, all studies (100%) were judged as having some concerns (Figure 2B). For treatment compliance, assessed in 11 studies, two (18.2%) were judged at high risk of bias, while the remaining nine (81.8%) were rated as having some concerns (Figure 2C).

### 3.4 Meta analysis

Variability in outcome reporting across the included studies resulted in differing numbers of studies contributing to each quantitative analysis. Therefore, analyses were conducted using studies with sufficient data for each specific outcome. The number of studies contributing to each outcome is detailed in the supplementary influence analysis tables (Supplementary Tables 7–14).

### 3.5 Eradication rate

**Figure 3:**
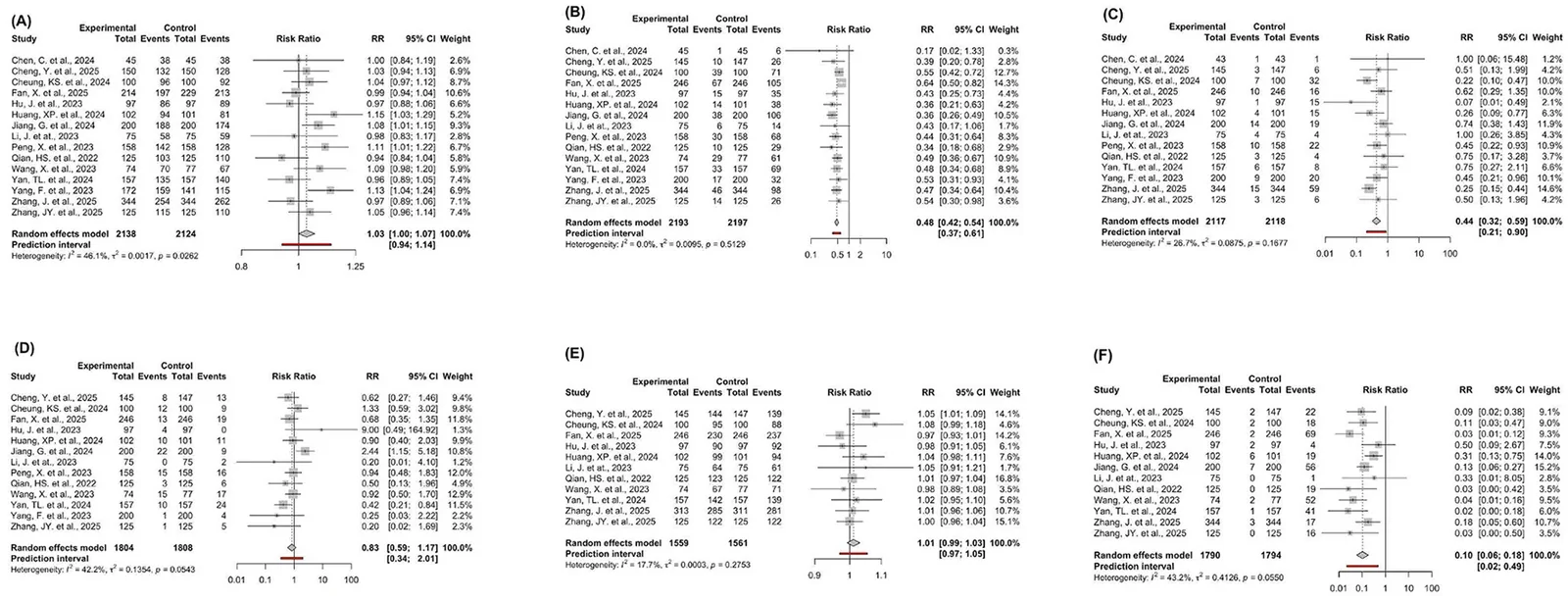
Forest plot of the meta-analysis: **(A)** eradication rate, **(B)** adverse events, **(C)** nausea, **(D)** diarrhea, **(E)** treatment compliance, **(F)** bitter taste.

**Figure 4:**
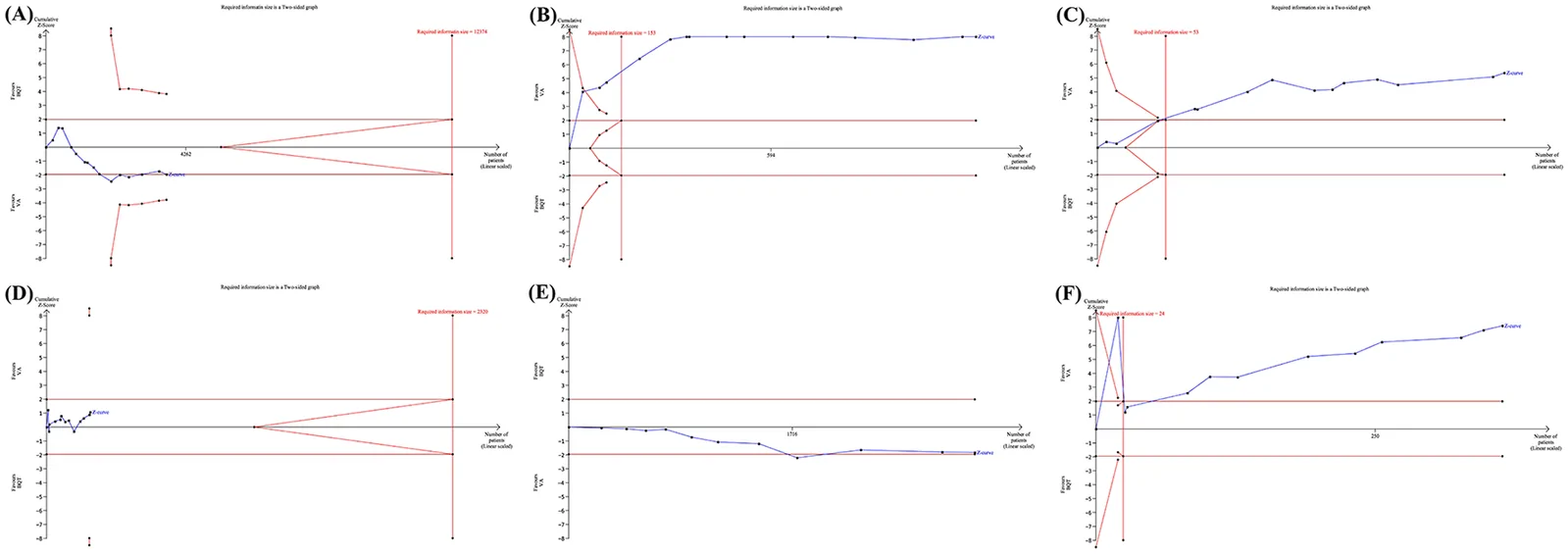
Trial sequential analysis of the outcomes: **(A)** eradication rate, **(B)** adverse events, **(C)** nausea, **(D)** diarrhea, **(E)** treatment compliance, **(F)** bitter taste.

In this meta-analysis, we incorporated 15 studies with a total sample size of 4,262 subjects, including 2,138 individuals treated with VA dual therapy and 2,124 receiving BQT. The crude pooled eradication rates were 86.4% (1,847/2,138) in the VA dual therapy group and 85.0% (1,806/2,124) in the BQT group. Using a random-effects model, the pooled RR was 1.03 (95% CI: 1.00–1.07; *p* = 0.05; *I*^2^ = 46.1%; Figure 3A). The funnel plot was symmetrical, and the Egger regression test was not statistically significant for publication bias (*p* = 0.84; Supplementary Figure 1A). Subgroup analyses revealed no statistically significant differences by intervention dosage (*p* = 0.13), amoxicillin frequency (*p* = 0.36), comparator-based regimens (*p* = 0.12), alcohol and smoking consumption status (*p* = 0.72), clarithromycin-based comparators (*p* = 0.13), or risk of bias (*p* = 0.65). However, subgroup analysis by treatment duration demonstrated statistically significant subgroup differences (*p* = 0.04), with 14-day regimens favoring VA dual therapy (RR 1.05, 95% CI: 1.01–1.08; *I*^2^ = 43.6%), whereas 10-day regimens showed no significant difference (RR 0.98, 95% CI: 0.93–1.03; *I*^2^ = 7.0%; Supplementary Table 7). Influence analysis did not identify any study as statistically influential (Supplementary Table 8). The TSA estimated the required information size (RIS) of 12,374 patients. The cumulative *Z*-curve remained within the monitoring boundaries, and the RIS was not reached, indicating that the current evidence remains insufficient to establish firm conclusions (Figure 4A).

### 3.6 Total adverse events

In this meta-analysis, we incorporated 15 studies with a total sample size of 4,390 subjects, comprising 2,193 individuals treated with VA dual therapy and 2,197 in the control group. The crude pooled rates of total adverse events were 17.1% (375/2,193) in the VA dual therapy group and 35.4% (778/2,197) in the BQT group. Using a random effects model, the pooled RR was 0.48 (95% CI: 0.42–0.54; *p* < 0.01; *I*^2^ = 0.0%; Figure 3B). The funnel plot demonstrated asymmetrical distribution, with the Egger's regression test suggesting potential publication bias (*p* = 0.04; Supplementary Figure 1B). Subgroup and influence analyses were deemed unnecessary, as no heterogeneity was observed. The TSA estimated a RIS of 153 patients. The cumulative *Z*-curve crossed both the conventional and trial sequential monitoring boundaries before reaching the RIS, indicating sufficient and robust evidence favoring the VA dual therapy group (Figure 4B).

### 3.7 Nausea

In this meta-analysis, we incorporated 14 studies with a total sample size of 4,235 subjects, comprising 2,117 individuals treated with VA dual therapy and 2,118 in the control group. The crude pooled nausea rates were 4.4% (93/2,117) in the VA dual therapy group and 10.6% (224/2,118) in the BQT group. Using a random effects model, the pooled RR was 0.44 (95% CI: 0.32–0.59; *p* < 0.01; *I*^2^ = 26.7%; Figure 3C). The funnel plot demonstrated a symmetrical distribution, and the Egger's regression test was not statistically significant for publication bias (*p* = 0.59; Supplementary Figure 1C). Subgroup analyses showed no statistically significant differences by intervention dosage (*p* = 0.48), amoxicillin frequency (*p* = 0.58), alcohol and smoking consumption status (*p* = 0.55), and treatment duration (*p* = 0.21). However, statistically significant subgroup differences were observed according to comparator-based regimens (*p* = 0.01) and clarithromycin-based comparators (*p* < 0.01; Supplementary Table 9). Influence analysis identified no statistically influential study (Supplementary Table 10). The TSA estimated the RIS of 53 patients. The cumulative *Z*-curve crossed the monitoring boundaries in favor of the VA dual therapy group, indicating firm evidence supporting a significant reduction in nausea with VA dual therapy (Figure 4C).

### 3.8 Diarrhea

In this meta-analysis, we incorporated 13 studies with a total sample size of 3,612 subjects, comprising 1,804 individuals treated with VA dual therapy and 1,808 in the control group. The crude pooled diarrhea rates were 6.7% (121/1,804) in the VA dual therapy group and 7.1% (128/1,808) in the BQT group. Using a random effects model, the pooled RR was 0.83 (95% CI: 0.59–1.17; *p* = 0.28; *I*^2^ = 42.2%; Figure 3D). The funnel plot did not reveal substantial asymmetry; however, the Egger's regression test demonstrated no significant publication bias (*p* = 0.52; Supplementary Figure 1D). Subgroup analyses showed no statistically significant difference by AMX dosage (*p* = 0.75), AMX frequency (*p* = 0.84), comparator-based regimens (*p* = 0.21), and clarithromycin-based comparator regimens (*p* = 0.20). However, a statistically significant difference was observed according to treatment duration (*p* = 0.02), with 10-day regimens favoring VA dual therapy (RR 0.49, 95% CI: 0.30–0.81), whereas 14-day regimens showed no significant difference (RR 1.00, 95% CI: 0.71–1.42; Supplementary Table 11). Influence analysis did not identify any study as significantly influential (Supplementary Table 12). The TSA estimated the RIS of 2,320 patients. However, the RIS was not reached, and the *Z*-curve remained within the monitoring boundaries without crossing the conventional significance threshold (Figure 4D).

### 3.9 Compliance

In this meta-analysis, we incorporated 11 studies with a sample size of 3,120 subjects, comprising 1,559 individuals treated with VA dual therapy and 1,561 in the control group. The crude pooled compliance rates were 93.5% (1,458/1,559) in the VA dual therapy group and 92.8% (1,449/1,561) in the BQT group. Using a random effects model, the pooled RR was 1.01 (95% CI: 0.99–1.03; *p* = 0.24; *I*^2^ = 17.7%; Figure 3E). The funnel plot revealed symmetry, with the Egger's regression test showing no significant publication bias (*p* = 0.51; Supplementary Figure 1E). Subgroup and influence analyses were deemed unnecessary, as low heterogeneity was observed. The TSA estimated a RIS of 15,559 patients. The *Z*-curve remained within the monitoring boundaries, and RIS was not reached (Figure 4E).

### 3.10 Bitter taste

In this meta-analysis, we incorporated 12 studies with a total sample size of 3,584 subjects, comprising 1,790 individuals treated with VA dual therapy and 1,794 in the control group. The crude pooled bitter taste rates were 5.6% (101/1,790) in the VA dual therapy group and 14.5% (260/1,794) in the BQT group. Using a random effects model, VA dual therapy was associated with a significantly lower risk of bitter taste compared with BQT (RR 0.10, 95% CI: 0.06–0.18; *p* < 0.01; *I*^2^ = 43.2%; Figure 3F). The funnel plot did not reveal substantial asymmetry, and Egger's regression test demonstrated no significant publication bias (*p* = 0.21; Supplementary Figure 1F). Subgroup analyses demonstrated a statistically significant difference according to AMX frequency (*p* = 0.02), with BID regimens (RR 0.03, 95% CI: 0.01–0.12) and QID regimens (RR 0.04, 95% CI: 0.01–0.13) showing lower risks of bitter taste compared with TID regimens (RR 0.15, 95% CI: 0.09–0.25). No statistically significant subgroup differences were observed according to AMX dosage (*p* = 0.64), comparator-based regimens (*p* = 0.26), clarithromycin-based comparator regimens (*p* = 0.07), or treatment duration (*p* = 0.17; Supplementary Table 13). Influence analysis did not identify any study as significantly influential (Supplementary Table 14). The TSA estimated a RIS of 24 patients. The cumulative *Z*-curve crossed both the conventional and TSA monitoring boundaries in favor of VA dual therapy, and the RIS was exceeded, indicating firm evidence for a reduced risk of bitter taste with VA dual therapy (Figure 4F).

We have summarized all these findings in Table 2.

**Table 2:** Meta-analysis outcomes of efficacy and safety for vonoprazan-amoxicillin dual therapy vs. bismuth-based quadruple therapy.

Variable studied	# of studies	Sample size	VA vs. BQT Pooled event rates (%)	Effect size RR (95% CI, I^2^)	p-value
ER	15	4262	86.4 vs. 85.0	1.03 (1.00–1.07, 46.1%)	0.05
Total AEs	**15**	**4390**	**17.1 vs. 35.4**	**0.48 (0.42**–**0.54, 0.0%)**	**< 0.01**
Nausea	**14**	**4235**	**4.4 vs. 10.6**	**0.44 (0.32**–**0.59, 26.7%)**	**< 0.01**
Diarrhea	13	3612	6.7 vs. 7.1	0.83 (0.59–1.17, 42.2%)	0.28
Compliance	11	3120	93.5 vs. 92.8	1.01 (0.99–1.03, 17.7%)	0.24
Bitter taste	**12**	**3584**	**5.6 vs. 14.5**	**0.10 (0.06–0.18, 43.2%)**	**< 0.01**

AEs, adverse events; BQT, bismuth-based quadruple therapy; ER, eradication rate; VA, vonoprazan-amoxicillin dual therapy. Bold values indicate statistically significant results (*p* < 0.05).

### 3.11 Assessment of the certainty of the evidence and summary of findings

According to the GRADE assessment, the certainty of evidence varied across outcomes. The certainty for eradication rate was rated as moderate due to inconsistency across studies despite the large sample size and relatively precise estimates. Total adverse events and nausea were supported by low-certainty evidence, primarily downgraded because of concerns regarding risk of bias. Bitter taste was also rated as low-certainty evidence despite showing a consistent reduction with VA dual therapy. The certainty of evidence for diarrhea was judged as very low because of combined concerns related to risk of bias, inconsistency, and imprecision. Compliance was supported by low-certainty evidence, mainly limited by risk of bias and imprecision (Supplementary Table 15).

### 3.12 Trial sequential analysis

For eradication rate, the *Z*-curve remained within the monitoring boundaries, and the RIS was not reached, indicating that the current evidence remains insufficient to confirm superiority of VA dual therapy over BQT. For total adverse events, nausea, and bitter taste, the cumulative *Z*-curves crossed both the conventional and TSA-adjusted monitoring boundaries in favor of VA dual therapy, and the RIS was reached or exceeded, indicating firm evidence that VA dual therapy is associated with a lower risk of these adverse events compared with BQT. For diarrhea and compliance, the *Z*-curves remained within the monitoring boundaries and the RIS was not reached, suggesting that the current evidence remains underpowered and inconclusive for these outcomes. Overall, TSA findings suggest that VA dual therapy demonstrates a superior safety profile compared with BQT, particularly regarding overall adverse events, nausea, and bitter taste. However, the available evidence remains insufficient to establish superiority in eradication of efficacy and compliance outcomes. Further large-scale randomized controlled trials are warranted to clarify the role of VA dual therapy as a first-line alternative to BQT (Figure 4).

## 4 Discussion

### 4.1 Main findings

This meta-analysis of 15 RCTs comprising 4,410 patients demonstrates that VA dual therapy achieves eradication rates comparable to PPI-based BQT (86.4% vs. 85.0%; RR 1.03, 95% CI 1.00–1.07; p = 0.05; *I*^2^ = 46.1%) while conferring a significantly more favorable tolerability profile. VA dual therapy conferred a significantly lower risk of total AEs (RR 0.48, 95% CI 0.42–0.54; *p* < 0.01; *I*^2^ = 0%), independently corroborated by TSA. The primary limitation of the efficacy estimate is its borderline statistical significance and moderate heterogeneity; TSA confirmed that the current evidence base remains underpowered to establish superiority or non-inferiority in the formal sense. The observed heterogeneity may be explained by differences across studies in VPZ dosage, amoxicillin dosing frequency, treatment duration, and antibiotic regimens. In addition, patient-related characteristics, including age distribution, prior treatment history, geographic region, and baseline disease characteristics, may have influenced treatment response and tolerability. Regional variations in antibiotic resistance prevalence may also have contributed to differences in eradication outcomes. By restricting all comparator arms to conventional PPI-based BQT, this analysis eliminates the pharmacological heterogeneity introduced in prior syntheses.

### 4.2 Comparison with prior literature

These efficacy findings are consistent with Zhou BG et al., who reported no significant difference in *Helicobacter pylori* eradication rates using similar restrictions (Zhou et al., 2024). In contrast, previous meta-analysis by (Li et al. 2025) and (Zhang et al. 2025c) reported superior VA efficacy. However, both (Li et al. 2025) and (Zhou et al. 2024) included BQT regimen using VPZ instead of a PPI, introducing heterogeneity given the stronger acid suppression (Mori and Suzuki, 2019; Shinozaki et al., 2016). To ensure consistency, our analysis included only PPI-based BQT with conventional antibiotics as the comparator. Accordingly, the 2024 ACG Clinical Guideline recommends optimized BQT as the preferred first-line regimen for treatment-naïve patients when antibiotic susceptibility is unknown, while conditionally endorsing VA dual therapy as an alternative (Chey et al., 2024).

Regarding safety, both qualitative and quantitative analyses consistently favored VA dual therapy, corroborating findings from (Li et al. 2025), (Zhang et al. 2025b), (Abosheaishaa et al. 2025), and (Zhou et al. 2024). For nausea, VA dual therapy significantly reduced risk compared with BQT, consistent with (Li et al. 2025), (Abosheaishaa et al. 2025), and (Zhou et al. 2024). Likewise, VA dual therapy was associated with a lower risk of bitter taste compared with BQT, consistent with findings from (Li et al. 2025) and (Zhou et al. 2024), Importantly, our study included a larger number of studies and greater overall sample size, thereby increasing statistical power and robustness of available evidence, as further supported by TSA. For diarrhea, neither qualitative nor quantitative analyses showed a significant difference between regimens, consistent with (Li et al. 2025), (Abosheaishaa et al. 2025), and (Zhou et al. 2024). Previous pharmacological and microbiome studies suggest that VA dual therapy may exert a lower impact on gut microbiota and exhibit reduced susceptibility to CYP2C19 polymorphisms, potentially contributing to a lower overall incidence of gastrointestinal AEs (Kornatowska et al., 2025).

### 4.3 Biological mechanisms

The favorable tolerability profile of VA dual therapy observed in this meta-analysis is mechanistically consistent with the pharmacological properties of VPZ as a PCAB. Unlike conventional PPIs, which require acid-dependent activation and are influenced by CYP2C19 metabolism, VPZ provides rapid, potent, and sustained acid suppression through reversible and pH-independent inhibition of the H^+^/K^+^-ATPase, with more consistent effects across CYP2C19 metabolizer genotypes (Shin and Kim, 2013; Jenkins et al., 2015; Sugimoto et al., 2020). In addition, VPZ shows no significant pharmacokinetic changes under fed conditions, suggesting efficacy largely independent of meal timing (Liu and Hahn, 2025). These pharmacokinetic and pharmacodynamic characteristics may contribute to improved gastrointestinal tolerability and treatment adherence. The absence of a significant difference in diarrhea rates between VA and BQT is biologically plausible, as diarrhea associated with BQT is primarily driven by the antibiotic components of the regimen rather than the acid-suppressive agent itself, a pattern also observed with other PCABs such as fexuprazan, which similarly preserve intestinal barrier integrity and demonstrate anti-inflammatory properties distinct from conventional PPIs (Lee et al., 2025; Ju et al., 2026; Jeon et al., 2025).

### 4.4 Clinical implications

According to the GRADE assessment, certainty of evidence was moderate for eradication, low for total AEs, nausea, and bitter taste, and very low to low for diarrhea and compliance. Compliance rates were high in both groups and did not differ significantly in our meta-analysis, consistent with (Abosheaishaa et al. 2025) and (Zhou et al. 2024); however, (Li et al. 2025) and (Zhang et al. 2025c) reported a statistically significant compliance advantage with VA dual therapy. Given the large pill burden, frequent gastrointestinal side effects, and tetracycline-related contraindications associated with BQT, VA dual therapy represents a more convenient and better tolerated alternative that may facilitate broader clinical use and improve real-world adherence. An emerging area of interest is PCAB-based bismuth quadruple therapy (PCAB-BQT). Although substituting VPZ for a conventional PPI within BQT is unlikely to substantially improve eradication rates over optimized PPI-BQT, the superior tolerability of PCABs may meaningfully reduce gastrointestinal adverse effects, improve adherence, and enhance treatment completion, warranting prospective trials to determine whether these advantages translate into clinically significant benefits (Lee et al., 2025; Ju et al., 2026; Jeon et al., 2025). Adequately powered, geographically diverse RCTs remain necessary to clarify the role of VA dual therapy as a first-line alternative to BQT.

### 4.5 Strengths and limitations

This study has notable strengths. It provides comprehensive synthesis comparing VA dual therapy with BQT for *Helicobacter pylori eradication*, incorporating both qualitative and quantitative analyses across efficacy, safety, and compliance outcomes. By including only RCTs, the analysis minimizes selection bias and enhances the validity between treatment regimens. A key methodological strength is the exclusion of trials in which the BQT control arm employed VPZ rather than a conventional PPI, eliminating a source of systematic heterogeneity present in all previously published meta-analyses on this comparison. The use of the GRADE approach further supported a structured and transparent evaluation of evidence of certainty across outcomes, contributing to robust and clinically meaningful conclusions. This study also has limitations. All included studies were conducted in China, which substantially limits the external validity of these findings. Antimicrobial resistance profiles, particularly clarithromycin resistance, which exceeds 20–30% in China, differ markedly from those in North America, Europe, Southeast Asia, and low-resource settings, where distinct resistance ecologies may yield different eradication outcomes. Additionally, the prevalence of CYP2C19 rapid metabolizers is considerably higher in East Asian populations than in Western cohorts, meaning the pharmacodynamic advantage of VPZ over PPIs may be more pronounced in the populations studied here than elsewhere. Because all included trials were conducted in China, findings should be interpreted cautiously in regions with different antimicrobial resistance patterns and host pharmacogenomic characteristics. The variations in treatment duration, dosages, and patient populations, may have contributed to residual heterogeneity and reduced the precision of pooled estimates. Publication bias could not be excluded for most outcomes, as the limited number of available studies precluded formal Egger's testing; furthermore, funnel plot asymmetry was observed for nausea and diarrhea, which may reflect publication bias, small-study effects, or genuine clinical heterogeneity. Language restrictions in the search further limited the available data, potentially affecting the robustness of conclusions. Finally, for future research, it may be valuable to include direct comparisons between VA dual therapy and VPZ-based triple therapy, as such studies could provide deeper insight into the relative advantages and clinical positioning of these regimens.

## 5 Conclusion

This systematic review and meta-analysis demonstrated that VA dual therapy achieved modest but borderline statistically significant improvements in *Helicobacter pylori* eradication rates compared to PPI-based BQT, however, TSA supported the robustness of the evidence. Moreover, VA dual therapy demonstrated a more favorable safety profile than conventional quadruple therapy, with significantly fewer total AE, particularly nausea and bitter taste. Although diarrhea, and compliance outcomes were comparable between regimens, the reduced adverse-event burden and simpler regimen of VA dual therapy may support better tolerability and adherence in clinical practice. Robust evidence supporting improved eradication rates and reduced adverse events suggests that VA dual therapy may represent a feasible therapeutic alternative that could broaden management options for *Helicobacter pylori* infection. Nevertheless, larger randomized controlled trials are needed to further evaluate compliance rates and long-term outcomes.

## Data Availability

The original contributions presented in the study are included in the article/Supplementary material, further inquiries can be directed to the corresponding authors.
