# Supplementary material for: Comparative efficacy and safety of vonoprazan-based dual therapy vs. bismuth-based quadruple therapy for Helicobacter pylori eradication: a GRADE-assessed systematic review and meta-analysis with trial sequential analysis
Source: Front Microbiol. 2026 Aug 24;17:1872669. doi: 10.3389/fmicb.2026.1872669 (PMC13547320; doi:10.3389/fmicb.2026.1872669)
Supplement: Supplementary file 1 [file Data_Sheet_1.docx]

**Comparative Efficacy and Safety of Vonoprazan-Based Dual Therapy Versus Bismuth-Based Quadruple Therapy for Helicobacter pylori Eradication: A GRADE-Assessed Systematic Review and Meta-Analysis with Trial Sequential Analysis**

¶Alejandro Chen Liang, MD†^1^; ¶Yeison Cruz Castillo, MD ^2^; Lorena M. Murrieta Bruciaga, MD ^3^; Salma Beltrán Covarrubias, MD ^4^; José García-Corella, MD^5^; Barbara Abreu Lopez, MD ^6^; Hector Jonan Flores Uribe, MD ^7^; Vanessa Pamela Salolin Vargas, MD ^8^; Gabriela Flores Monar, MD^9^; Ishaan Kalha, MD^10^; Ernesto Calderón-Martínez, MD†^11^

^1^Centro de Estudios Universitarios Xochicalco Campus Tijuana, Tijuana, México

^2^Universidad Autónoma de Santo Domingo, Santo Domingo, República Dominicana

^3^Universidad del Valle de México, Hermosillo, México

^4^Universidad de Guadalajara, Guadalajara, México

^5^Department of Medicine, UCLA-Kern Medical, Bakersfield, CA y David Geffen School of Medicine at UCLA, Los Angeles, CA.

^6^Maimonides Medical Center, New York, United States

^7^Centro de Estudios Universitarios Xochicalco Campus Tijuana, Tijuana, México

^8^Universidad Westhill, Facultad de Medicina, Ciudad de México, México

^9^University of Miami/Jackson Health System, Miami, Florida, USA

^10^Division of Gastroenterology, Department of Medicine, UCLA-Kern Medical, Bakersfield, CA y David Geffen School of Medicine at UCLA, Los Angeles, CA.

^11^Texas Tech University Health Sciences Center El Paso, El Paso, USA

^1^[alex95chenliang@gmail.com](mailto:alex95chenliang@gmail.com) | ^2^ [yeisoncc_159@hotmail.com](mailto:4yeisoncc_159@hotmail.com) | ^3^lorena.murrieta85@gmail.com  ^4^[salmabeltranmd@gmail.com](mailto:salmabeltranmd@gmail.com) | ^5^[drgarciacorella@gmail.com](mailto:drgarciacorella@gmail.com)  ^6^Barbi.abreu90@gmail.com | [^7^jonan0928@gmail.com](mailto:7jonan0928@gmail.com) |^8^[vanesalolin@gmail.com](mailto:vanesalolin@gmail.com) | [^9^gaby_91v@hotmail.com](mailto:9gaby_91v@hotmail.com) | | ^10^Ishaan.kalha@kernmedical.com | ^1^ernestocalderon.mtz@gmail.com

**ORCID:** ^1^ 0009-0005-6769-7505 | ^2^ 0009-0005-3660-1304| ^3^ 0009-0005-1317-7379 | ^4^0009-0006-7390-1397 |

^5^0009-0003-3592-9039 | ^4^ 0009-0008-7289-4839 | ^5^ 0000-0002-7343-0023 | ^6^ 0009-0007-6863-5300 | ^9^ 0000-0001-6115-3113 | ^11^ 0000-0003-2237-9536

†Corresponding Authors:

Alejandro Chen Liang, MD ([alex95chenliang@gmail.com](mailto:alex95chenliang@gmail.com))

Ernesto Calderón Martinez, MD ([ernestocalderon.mtz@gmail.com](mailto:ernestocalderon.mtz@gmail.com))

¶These authors shared first authorship.

**Supplementary material index**

[**Supplementary table 1. Search in PubMed	2**](#_Toc1610174770)

[**Supplementary table 2. Search in Embase	3**](#_Toc201205264)

[**Supplementary table 3. Search in Web of science	3**](#_Toc113074553)

[**Supplementary table 4. Search in Google Schoolar	4**](#_Toc1504447486)

[**Supplementary table 5. Search in CINAHL	4**](#_Toc139306904)

[**Supplementary table 6. Search in Cochrane	4**](#_Toc674278962)

[**Supplementary table 7. Subgroup Analysis: Eradication Rate	4**](#_Toc2088443249)

[**Supplementary table 8. Influence Analysis: Eradication Rate	5**](#_Toc390924384)

[**Supplementary table 9. Subgroup Analysis: Nausea	5**](#_Toc2078072440)

[**Supplementary table 10. Influence Analysis: Nausea	5**](#_Toc1382171926)

[**Supplementary table 11. Subgroup Analysis: Diarrhea	6**](#_Toc1918462940)

[**Supplementary table 12. Influence Analysis: Diarrhea	6**](#_Toc1998753754)

[**Supplementary table 13. Influence Analysis: Bitter taste	7**](#_Toc1430489714)

[**Supplementary table 14. Influence Analysis: Bitter taste	7**](#_Toc25131342)

[**Supplementary table 13. GRADE assessment	7**](#_Toc313865778)

[**Supplementary Figures 1. Funnel Plot of the Meta-Analysis Outcomes	8**](#_Toc1079373570)

[**PRISMA 2020 Checklist	9**](#_Toc539694485)

[**PRISMA 2020 Abstract Checklist	12**](#_Toc997806895)

# **Supplementary table 1.** Search in PubMed

| Boolean search | Results |
| --- | --- |
| (("Helicobacter pylori"[MeSH Terms]) OR ("Helicobacter pylori"[Title/Abstract]) OR ("H. pylori"[Title/Abstract]) OR ("Helicobacter infection"[Title/Abstract]) OR ("Helicobacter infections"[Title/Abstract]))  AND  ((Vonoprazan[MeSH Terms]) OR (vonoprazan[Title/Abstract]) OR ("potassium-competitive acid blocker"[Title/Abstract]) OR (PCAB[Title/Abstract]))  AND  ((amoxicillin[MeSH Terms]) OR (amoxicillin[Title/Abstract]))  AND  (("Bismuth quadruple therapy"[Title/Abstract]) OR ("quadruple therapy"[Title/Abstract]) OR (bismuth[Title/Abstract] AND tetracycline[Title/Abstract] AND metronidazole[Title/Abstract] AND ("proton pump inhibitor"[Title/Abstract] OR PPI[Title/Abstract])))  AND  ((eradication[Title/Abstract]) OR ("treatment outcome"[MeSH Terms]) OR ("treatment outcome"[Title/Abstract]) OR ("adverse events"[Title/Abstract]) OR ("treatment compliance"[Title/Abstract]) OR (compliance[Title/Abstract]) OR (resistance[Title/Abstract]) OR ("antibiotic resistance"[Title/Abstract]) OR ("regional resistance"[Title/Abstract]) OR ("strain-specific resistance"[Title/Abstract])) | 92 |

# **Supplementary table 2.** Search in Embase

| Boolean search | Results |
| --- | --- |
| ('helicobacter pylori':ti,ab OR 'h. pylori':ti,ab OR 'hp infection':ti,ab OR 'h pylori infection':ti,ab OR 'helicobacter infection':ti,ab)  AND  ('vonoprazan':ti,ab OR 'potassium-competitive acid blocker':ti,ab OR 'pcab':ti,ab)  AND  ('amoxicillin':ti,ab)  AND  ('dual therapy':ti,ab OR 'va regimen':ti,ab OR 'vonoprazan amoxicillin':ti,ab OR 'vonoprazan and amoxicillin':ti,ab)  AND  ('bismuth quadruple therapy':ti,ab OR 'bqt':ti,ab OR 'quadruple therapy':ti,ab OR 'b-quadruple':ti,ab OR 'proton pump inhibitor':ti,ab OR 'ppi':ti,ab OR 'tetracycline':ti,ab OR 'metronidazole':ti,ab)  AND  ('eradication':ti,ab OR 'eradication rate':ti,ab OR 'treatment outcome':ti,ab OR 'adverse event':ti,ab OR 'safety':ti,ab OR 'compliance':ti,ab OR 'antibiotic resistance':ti,ab) | 151 |

# **Supplementary table 3.** Search in Web of science

| Boolean search | Results |
| --- | --- |
| TS=("Helicobacter pylori" OR "H. pylori" OR "Hp infection" OR "H pylori infection" OR "Helicobacter infection")  AND  TS=("vonoprazan" OR "potassium-competitive acid blocker" OR "PCAB")  AND  TS=("amoxicillin")  AND  TS=("dual therapy" OR "VA regimen" OR "vonoprazan amoxicillin")  AND  TS=("bismuth quadruple therapy" OR "BQT" OR "quadruple therapy" OR "B-quadruple" OR "proton pump inhibitor" OR "PPI" OR "tetracycline" OR "metronidazole")  AND  TS=("eradication" OR "eradication rate" OR "treatment outcome") | 25,975 |

# **Supplementary table 4.** Search in Google Schoolar

| Boolean search | Results |
| --- | --- |
| ("Helicobacter pylori" OR "H. pylori") AND  ("vonoprazan" AND "amoxicillin" OR "dual therapy") AND  ("bismuth quadruple therapy" OR "BQT" OR ("PPI" AND "bismuth" AND "tetracycline" AND "metronidazole")) AND  ("eradication rate" OR "treatment success" OR "adverse events" OR "compliance" OR "antibiotic resistance") | 886 |

# **Supplementary table 5.** Search in CINAHL

| Boolean search | Results |
| --- | --- |
| ("Helicobacter pylori" OR "H. pylori" OR "H pylori" OR Hpylori)  AND  (vonoprazan OR "potassium-competitive acid blocker" OR PCAB)  AND  (amoxicillin)  AND  ("dual therapy" OR "vonoprazan and amoxicillin" OR VA OR "vonoprazan dual therapy")  AND  ("bismuth quadruple therapy" OR BQT OR "bismuth quadruple" OR "bismuth-based quadruple therapy" OR "bismuth quadruple therapy" OR "standard therapy") | 3 |

# **Supplementary table 6.** Search in Cochrane

| **Boolean search** | **Results** |
| --- | --- |
| ("Helicobacter pylori" OR "H. pylori" OR "H pylori" OR Hpylori) AND (vonoprazan OR "potassium-competitive acid blocker" OR PCAB) AND (amoxicillin) AND ("dual therapy" OR "vonoprazan and amoxicillin" OR VA OR "vonoprazan dual therapy") AND ("bismuth quadruple therapy" OR BQT OR "bismuth quadruple" OR "bismuth-based quadruple therapy" OR "standard therapy") | 396 |

# **Supplementary table 7.** Subgroup Analysis: Eradication Rate

| **Variable** | **Subgroup** | **k** | **RR (95% CI)** | **I² (%)** | **p-value (Between Groups)** |
| --- | --- | --- | --- | --- | --- |
| Risk of Bias | Low | 12 | 1.02 (0.99–1.06) | 29.90 | 0.65 |
|  | Some concerns | 3 | 1.05 (0.95–1.17) | 74.90 |  |
| AMX Frequency | TID | 11 | 1.04 (1.00–1.07) | 40.10 | 0.36 |
|  | BID | 1 | 0.99 (0.94–1.04) | — |  |
|  | QID | 3 | 1.04 (0.94–1.16) | 69.20 |  |
| Comparator-Based Regimens | AMX+FZD | 2 | 0.99 (0.88–1.12) | 0.00 | 0.12 |
|  | AMX+CLA | 9 | 1.04 (1.00–1.08) | 53.70 |  |
|  | TET+NIT | 1 | 1.04 (0.97–1.12) | — |  |
|  | AMX+NIT | 1 | 0.97 (0.88–1.06) | — |  |
|  | NIT+CLA | 1 | 1.13 (1.04–1.24) | — |  |
|  | FZD+TET | 1 | 0.97 (0.89–1.06) | — |  |
| Alcohol Consumption /Smoking | No | 1 | 1.00 (0.84–1.19) | — | 0.72 |
|  | Yes | 14 | 1.03 (1.00–1.07) | 49.70 |  |
| Clarithromycin-Based Comparators | No | 5 | 1.00 (0.95–1.05) | 0.00 | 0.13 |
|  | Yes | 10 | 1.05 (1.01–1.09) | 57.30 |  |
| Treatment Duration (days) | 14 | 12 | 1.05 (1.01–1.08) | 43.60 | 0.045 |
|  | 10 | 3 | 0.98 (0.93–1.03) | 7.00 |  |
| AMX Dose (mg) | 1000 | 12 | 1.02 (0.99–1.06) | 49.50 | 0.13 |
|  | 750 | 3 | 1.08 (1.02–1.15) | 0.00 |  |

# **Supplementary table 8.** Influence Analysis: Eradication Rate

| **Author** | **Effect (95% CI: Lower; Upper)** | **I^2** | **DFFITS** | **Cook's D** | **QE (del)** | **Is Influential** |
| --- | --- | --- | --- | --- | --- | --- |
| Omitting Chen, C. et al., 2024 | 1.03 (1.00; 1.07) | 49.72 | -0.05 | 0.00 | 25.85 | No |
| Omitting Cheng, Y. et al., 2025 | 1.03 (1.00; 1.07) | 49.93 | -0.01 | 0.00 | 25.96 | No |
| Omitting Cheung, KS. et al., 2024 | 1.03 (1.00; 1.07) | 49.66 | 0.06 | 0.00 | 25.83 | No |
| Omitting Fan, X. et al., 2025 | 1.04 (1.00; 1.07) | 44.17 | -0.30 | 0.09 | 23.29 | No |
| Omitting Hu, J. et al., 2023 | 1.04 (1.00; 1.07) | 45.88 | -0.29 | 0.08 | 24.02 | No |
| Omitting Huang, XP. et al., 2024 | 1.03 (0.99; 1.06) | 41.41 | 0.37 | 0.13 | 22.19 | No |
| Omitting Jiang, G. et al., 2024 | 1.03 (0.99; 1.06) | 44.83 | 0.29 | 0.08 | 23.56 | No |
| Omitting Li, J. et at., 2023 | 1.03 (1.00; 1.07) | 49.35 | -0.09 | 0.01 | 25.67 | No |
| Omitting Peng, X. et al., 2023 | 1.03 (0.99; 1.06) | 44.27 | 0.32 | 0.10 | 23.32 | No |
| Omitting Qian, HS. et al., 2022 | 1.04 (1.01; 1.07) | 42.33 | -0.37 | 0.13 | 22.54 | No |
| Omitting Wang, X. et al., 2023 | 1.03 (1.00; 1.06) | 47.69 | 0.20 | 0.04 | 24.85 | No |
| Omitting Yan, TL. et al., 2024 | 1.04 (1.00; 1.07) | 44.35 | -0.34 | 0.11 | 23.36 | No |
| Omitting Yang, F. et al., 2023 | 1.02 (0.99; 1.06) | 38.98 | 0.46 | 0.18 | 21.31 | No |
| Omitting Zhang, J. et al., 2025 | 1.04 (1.00; 1.07) | 45.65 | -0.30 | 0.09 | 23.92 | No |
| Omitting Zhang, JY. et al., 2025 | 1.03 (1.00; 1.07) | 49.68 | 0.06 | 0.00 | 25.84 | No |

# **Supplementary table 9.** Subgroup Analysis: Nausea

| **Variable** | **Subgroup** | **k** | **RR (95% CI)** | **I² (%)** | **p-value (Between Groups)** |
| --- | --- | --- | --- | --- | --- |
| AMX Frequency | TID | 11 | 0.41 (0.28–0.59) | 36.80 | 0.58 |
|  | BID | 1 | 0.63 (0.29–1.35) | — |  |
|  | QID | 2 | 0.50 (0.26–0.95) | 0.00 |  |
| Comparator-Based Regimens | AMX+FZD | 2 | 1.00 (0.30–3.35) | 0.00 | 0.01 |
|  | AMX+CLA | 8 | 0.56 (0.40–0.78) | 0.00 |  |
|  | TET+NIT | 1 | 0.22 (0.10–0.47) | — |  |
|  | AMX+NIT | 1 | 0.07 (0.01–0.49) | — |  |
|  | NIT+CLA | 1 | 0.45 (0.21–0.96) | — |  |
|  | FZD+TET | 1 | 0.25 (0.15–0.44) | — |  |
| Alcohol/Smoking Consumption | No | 1 | 1.00 (0.06–15.48) | — | 0.55 |
|  | Yes | 13 | 0.43 (0.32–0.59) | 30.90 |  |
| Clarithromycin-Based Comparators | No | 5 | 0.27 (0.18–0.41) | 40.30 | 0.007 |
|  | Yes | 9 | 0.54 (0.40–0.73) | 0.00 |  |
| Treatment Duration (days) | 14 | 11 | 0.41 (0.29–0.57) | 36.50 | 0.21 |
|  | 10 | 3 | 0.67 (0.33–1.38) | 0.00 |  |
| AMX Dose (mg) | 1000 | 12 | 0.42 (0.30–0.59) | 31.50 | 0.48 |
|  | 750 | 2 | 0.54 (0.28–1.04) | 2.50 |  |

# **Supplementary table 10.** Influence Analysis: Nausea

| **Author** | **Effect (95% CI: Lower; Upper)** | **I^2** | **DFFITS** | **Cook's D** | **QE (del)** | **Is Influential** |
| --- | --- | --- | --- | --- | --- | --- |
| Omitting Chen, C. et al., 2024 | 0.43 (0.32; 0.59) | 30.87 | 0.06 | 0.00 | 17.36 | No |
| Omitting Cheng, Y. et al., 2025 | 0.43 (0.32; 0.60) | 32.09 | 0.03 | 0.00 | 17.67 | No |
| Omitting Cheung, KS. et al., 2024 | 0.47 (0.35; 0.64) | 17.66 | -0.49 | 0.22 | 14.57 | No |
| Omitting Fan, X. et al., 2025 | 0.42 (0.30; 0.58) | 27.97 | 0.24 | 0.06 | 16.66 | No |
| Omitting Hu, J. et al., 2023 | 0.45 (0.33; 0.61) | 16.75 | -0.25 | 0.06 | 14.41 | No |
| Omitting Huang, XP. et al., 2024 | 0.45 (0.33; 0.62) | 29.16 | -0.23 | 0.05 | 16.94 | No |
| Omitting Jiang, G. et al., 2024 | 0.40 (0.30; 0.55) | 18.11 | 0.57 | 0.27 | 14.65 | No |
| Omitting Li, J. et at., 2023 | 0.42 (0.31; 0.57) | 25.62 | 0.24 | 0.06 | 16.13 | No |
| Omitting Peng, X. et al., 2023 | 0.44 (0.31; 0.61) | 32.19 | 0.00 | 0.00 | 17.70 | No |
| Omitting Qian, HS. et al., 2022 | 0.43 (0.31; 0.58) | 30.03 | 0.13 | 0.02 | 17.15 | No |
| Omitting Yan, TL. et al., 2024 | 0.42 (0.31; 0.58) | 27.31 | 0.25 | 0.06 | 16.51 | No |
| Omitting Yang, F. et al., 2023 | 0.44 (0.31; 0.61) | 32.25 | -0.01 | 0.00 | 17.71 | No |
| Omitting Zhang, J. et al., 2025 | 0.48 (0.36; 0.65) | 11.34 | -0.74 | 0.37 | 13.54 | No |
| Omitting Zhang, JY. et al., 2025 | 0.43 (0.32; 0.60) | 32.13 | 0.03 | 0.00 | 17.68 | No |

# **Supplementary table 11.** Subgroup Analysis: Diarrhea

| **Subgroup Variable** | **Subgroup** | **k** | **RR (95% CI)** | **I² (%)** | **p-value (Between Groups)** |
| --- | --- | --- | --- | --- | --- |
| **AMX Frequency** | TID | 9 | 0.83 (0.46–1.48) | 59.2 | 0.840 |
|  | BID | 1 | 0.68 (0.35–1.35) | — |  |
|  | QID | 3 | 0.87 (0.57–1.34) | 0.0 |  |
| **Comparator-Based Regimens** | AMX+CLA | 9 | 0.80 (0.55–1.16) | 46.2 | 0.207 |
|  | TET+NIT | 1 | 1.33 (0.59–3.02) | — |  |
|  | AMX+NIT | 1 | 9.00 (0.49–164.92) | — |  |
|  | AMX+FZD | 1 | 0.20 (0.01–4.10) | — |  |
|  | NIT+CLA | 1 | 0.25 (0.03–2.22) | — |  |
| **Clarithromycin-Based Comparator** | Yes | 10 | 0.77 (0.53–1.12) | 43.8 | 0.199 |
|  | No | 3 | 1.35 (0.63–2.89) | 36.9 |  |
| **Treatment Duration (days)** | 10 | 3 | 0.49 (0.30–0.81) | 0.0 | 0.023 |
|  | 14 | 10 | 1.00 (0.71–1.42) | 37.1 |  |
| **AMX Dose (mg)** | 1000 | 10 | 0.81 (0.51–1.28) | 54.3 | 0.754 |
|  | 750 | 3 | 0.90 (0.57–1.40) | 0.0 |  |

# **Supplementary table 12.** Influence Analysis: Diarrhea

| **Author** | **Effect (95% CI: Lower; Upper)** | **I^2** | **DFFITS** | **Cook's D** | **QE (del)** | **Is Influential** |
| --- | --- | --- | --- | --- | --- | --- |
| Omitting Cheng, Y. et al., 2025 | 0.85 (0.59; 1.24) | 45.52 | -0.14 | 0.02 | 20.19 | No |
| Omitting Cheung, KS. et al., 2024 | 0.79 (0.54; 1.14) | 43.48 | 0.30 | 0.10 | 19.46 | No |
| Omitting Fan, X. et al., 2025 | 0.85 (0.58; 1.24) | 45.82 | -0.10 | 0.01 | 20.30 | No |
| Omitting Hu, J. et al., 2023 | 0.80 (0.57; 1.13) | 39.54 | 0.18 | 0.03 | 18.19 | No |
| Omitting Huang, XP. et al., 2024 | 0.82 (0.56; 1.20) | 46.92 | 0.09 | 0.01 | 20.72 | No |
| Omitting Jiang, G. et al., 2024 | 0.75 (0.57; 0.97) | 9.76 | 0.84 | 0.36 | 12.19 | No |
| Omitting Li, J. et at., 2023 | 0.84 (0.60; 1.19) | 44.60 | -0.10 | 0.01 | 19.86 | No |
| Omitting Peng, X. et al., 2023 | 0.81 (0.55; 1.20) | 46.72 | 0.13 | 0.02 | 20.65 | No |
| Omitting Qian, HS. et al., 2022 | 0.85 (0.60; 1.21) | 45.40 | -0.14 | 0.02 | 20.14 | No |
| Omitting Wang, X. et al., 2023 | 0.81 (0.55; 1.20) | 46.78 | 0.12 | 0.02 | 20.67 | No |
| Omitting Yan, TL. et al., 2024 | 0.92 (0.67; 1.27) | 32.35 | -0.71 | 0.37 | 16.26 | No |
| Omitting Yang, F. et al., 2023 | 0.85 (0.60; 1.20) | 43.65 | -0.16 | 0.02 | 19.52 | No |
| Omitting Zhang, JY. et al., 2025 | 0.86 (0.61; 1.21) | 41.97 | -0.20 | 0.04 | 18.95 | No |

# **Supplementary table 13.** Influence Analysis: Bitter taste

| **Variable** | **Subgroup** | **k** | **RR (95% CI)** | **I² (%)** | **p-value (Between Groups)** |
| --- | --- | --- | --- | --- | --- |
| **AMX Frequency** | TID | 9 | 0.15 (0.09–0.25) | 22.5 | 0.017 |
|  | BID | 1 | 0.03 (0.01–0.12) | — |  |
|  | QID | 2 | 0.04 (0.01–0.13) | 0.0 |  |
| **Comparator-Based Regimens** | AMX+CLA | 8 | 0.07 (0.03–0.15) | 53.8 | 0.260 |
|  | TET+NIT | 1 | 0.11 (0.03–0.47) | — |  |
|  | AMX+NIT | 1 | 0.50 (0.09–2.67) | — |  |
|  | AMX+FZD | 1 | 0.33 (0.01–8.05) | — |  |
|  | FZD+TET | 1 | 0.18 (0.05–0.60) | — |  |
| **Alcohol Consumption/Smoking** | Yes | 12 | 0.10 (0.06–0.18) | 43.2 | — |
| **Clarithromycin-Based Comparator** | Yes | 8 | 0.07 (0.03–0.15) | 53.8 | 0.067 |
|  | No | 4 | 0.20 (0.09–0.44) | 0.0 |  |
| **Treatment Duration (days)** | 10 | 3 | 0.05 (0.02–0.15) | 0.0 | 0.171 |
|  | 14 | 9 | 0.12 (0.06–0.24) | 47.6 |  |
| **AMX Dose (mg)** | 1000 | 10 | 0.11 (0.06–0.20) | 45.2 | 0.636 |
|  | 750 | 2 | 0.07 (0.01–0.43) | 30.3 |  |

# **Supplementary table 14.** Influence Analysis: Bitter taste

| **Author** | **Effect (95% CI: Lower; Upper)** | **I^2** | **DFFITS** | **Cook's D** | **QE (del)** | **Is Influential** |
| --- | --- | --- | --- | --- | --- | --- |
| Omitting Cheng, Y. et al., 2025 | 0.10 (0.05; 0.19) | 47.99 | 0.01 | 0.00 | 19.23 | No |
| Omitting Cheung, KS. et al., 2024 | 0.10 (0.05; 0.19) | 48.32 | 0.08 | 0.01 | 19.35 | No |
| Omitting Fan, X. et al., 2025 | 0.12 (0.07; 0.21) | 33.79 | -0.60 | 0.30 | 15.10 | No |
| Omitting Hu, J. et al., 2023 | 0.09 (0.05; 0.16) | 38.89 | 0.41 | 0.16 | 16.36 | No |
| Omitting Huang, XP. et al., 2024 | 0.09 (0.05; 0.15) | 26.06 | 0.65 | 0.27 | 13.52 | No |
| Omitting Jiang, G. et al., 2024 | 0.10 (0.05; 0.19) | 48.28 | 0.17 | 0.04 | 19.33 | No |
| Omitting Li, J. et at., 2023 | 0.10 (0.06; 0.18) | 47.22 | 0.13 | 0.02 | 18.95 | No |
| Omitting Qian, HS. et al., 2022 | 0.11 (0.06; 0.19) | 45.00 | -0.18 | 0.03 | 18.18 | No |
| Omitting Wang, X. et al., 2023 | 0.12 (0.06; 0.21) | 40.30 | -0.38 | 0.14 | 16.75 | No |
| Omitting Yan, TL. et al., 2024 | 0.11 (0.06; 0.20) | 40.41 | -0.35 | 0.12 | 16.78 | No |
| Omitting Zhang, J. et al., 2025 | 0.10 (0.05; 0.18) | 47.11 | 0.25 | 0.07 | 18.91 | No |
| Omitting Zhang, JY. et al., 2025 | 0.11 (0.06; 0.19) | 45.73 | -0.15 | 0.02 | 18.43 | No |

# **Supplementary table 15. GRADE assessment**

| **Certainty assessment** | | | | | | | **№ of patients** | | **Effect** | | **Certainty** | **Importance** |
| --- | --- | --- | --- | --- | --- | --- | --- | --- | --- | --- | --- | --- |
| **№ of studies** | **Study design** | **Risk of bias** | **Inconsistency** | **Indirectness** | **Imprecision** | **Other considerations** | **Vonoprazan-Based Dual Therapy** | **Bismuth-Based Quadruple Therapy** | **Relative** **(95% CI)** | **Absolute** **(95% CI)** |  |  |
| **Eradication rate** | | | | | | | | | | | | |
| 15 | randomised trials | not serious | serious^a^ | not serious | not serious | none | 1867/2138 (87.3%) | 1806/2124 (85.0%) | **RR 1.03** (0.99 to 1.06) | **26 more per 1,000** (from 9 fewer to 51 more) | ⨁⨁⨁◯ Moderate^a^ | CRITICAL |
| **Adverse Events** | | | | | | | | | | | | |
| 15 | randomised trials | very serious^b^ | not serious | not serious | not serious | none | 369/2193 (16.8%) | 784/2197 (35.7%) | **RR 0.47** (0.42 to 0.53) | **189 fewer per 1,000** (from 207 fewer to 168 fewer) | ⨁⨁◯◯ Low^b^ | CRITICAL |
| **Nausea** | | | | | | | | | | | | |
| 14 | randomised trials | very serious^b^ | not serious | not serious | not serious | none | 90/2117 (4.3%) | 226/2118 (10.7%) | **RR 0.43** (0.32 to 0.59) | **61 fewer per 1,000** (from 73 fewer to 44 fewer) | ⨁⨁◯◯ Low^b^ | IMPORTANT |
| **Diarrhea** | | | | | | | | | | | | |
| 13 | randomised trials | very serious^b^ | serious^a^ | not serious | serious^c^ | none | 114/1804 (6.3%) | 135/1808 (7.5%) | **RR 0.82** (0.58 to 1.16) | **13 fewer per 1,000** (from 31 fewer to 12 more) | ⨁◯◯◯ Very low^a,b,c^ | IMPORTANT |
| **Compliance** | | | | | | | | | | | | |
| 11 | randomised trials | very serious^b^ | not serious | not serious | not serious^c^ | none | 1461/1559 (93.7%) | 1439/1561 (92.2%) | **RR 1.01** (0.99 to 1.03) | **9 more per 1,000** (from 9 fewer to 28 more) | ⨁⨁◯◯ Low^b,c^ | CRITICAL |
| **Bitter taste** | | | | | | | | | | | | |
| 12 | randomised trials | very serious^b^ | not serious | not serious | not serious | none | 26/1790 (1.5%) | 334/1794 (18.6%) | **RR 0.10** (0.05 to 0.18) | **168 fewer per 1,000** (from 177 fewer to 153 fewer) | ⨁⨁◯◯ Low^b^ | IMPORTANT |

**CI:** confidence interval; **RR:** risk ratio

#### **Explanations**

a. High I² values indicate substantial heterogeneity

b. A high risk of bias was identified more frequently among the included studies

c. The result is not statistically significant, and the Cl is wide

# **Supplementary Figures 1.** Funnel Plot of the Meta-Analysis Outcomes

**Supplementary Figures 1.** Forest plot of the meta-analysis: A) Eradication Rate; B) Adverse Events; C) Nausea; D) Diarrhea; E) Compliance; F) Bitter Taste

# **PRISMA 2020 Checklist**

| **Section and Topic** | **Item #** | **Checklist item** | **Location where item is reported** |
| --- | --- | --- | --- |
| **TITLE** | | |  |
| Title | 1 | Identify the report as a systematic review. | Page 1 |
| **ABSTRACT** | | |  |
| Abstract | 2 | See the PRISMA 2020 for Abstracts checklist. | Page 2-3 |
| **INTRODUCTION** | | |  |
| **Rationale** | 3 | Describe the rationale for the review in the context of existing knowledge. | Page 4 |
| **Objectives** | 4 | Provide an explicit statement of the objective(s) or question(s) the review addresses. | Page 5-8 |
| **METHODS** | | |  |
| **Eligibility criteria** | 5 | Specify the inclusion and exclusion criteria for the review and how studies were grouped for the syntheses. | Page 5 |
| **Information sources** | 6 | Specify all databases, registers, websites, organizations, reference lists and other sources searched or consulted to identify studies. Specify the date when each source was last searched or consulted. | Page 6-7 |
| **Search strategy** | 7 | Present the full search strategies for all databases, registers and websites, including any filters and limits used. | Page 7 |
| **Selection process** | 8 | Specify the methods used to decide whether a study met the inclusion criteria of the review, including how many reviewers screened each record and each report retrieved, whether they worked independently, and if applicable, details of automation tools used in the process. | Page 7-8 |
| **Data collection process** | 9 | Specify the methods used to collect data from reports, including how many reviewers collected data from each report, whether they worked independently, any processes for obtaining or confirming data from study investigators, and if applicable, details of automation tools used in the process. | Page 6 |
| **Data items** | 10a | List and define all outcomes for which data were sought. Specify whether all results that were compatible with each outcome domain in each study were sought (e.g. for all measures, time points, analyses), and if not, the methods used to decide which results to collect. | Page 7 |
|  | 10b | List and define all other variables for which data were sought (e.g. participant and intervention characteristics, funding sources). Describe any assumptions made about any missing or unclear information. | Page 6-8 |
| **Study risk of bias assessment** | 11 | Specify the methods used to assess risk of bias in the included studies, including details of the tool(s) used, how many reviewers assessed each study and whether they worked independently, and if applicable, details of automation tools used in the process. | Page 8-9 |
| **Effect measures** | 12 | Specify for each outcome the effect measure(s) (e.g. risk ratio, mean difference) used in the synthesis or presentation of results. | Page 8-9 |
| **Synthesis methods** | 13a | Describe the processes used to decide which studies were eligible for each synthesis (e.g. tabulating the study intervention characteristics and comparing against the planned groups for each synthesis (item #5)). | Page 9 |
|  | 13b | Describe any methods required to prepare the data for presentation or synthesis, such as handling of missing summary statistics, or data conversions. | Page 9 |
|  | 13c | Describe any methods used to tabulate or visually display results of individual studies and syntheses. | Page 9 |
|  | 13d | Describe any methods used to synthesize results and provide a rationale for the choice(s). If meta-analysis was performed, describe the model(s), method(s) to identify the presence and extent of statistical heterogeneity, and software package(s) used. | Page 9 |
|  | 13e | Describe any methods used to explore possible causes of heterogeneity among study results (e.g. subgroup analysis, meta-regression). | Page 9 |
|  | 13f | Describe any sensitivity analyses conducted to assess robustness of the synthesized results. | Page 9 |
| **Reporting bias assessment** | 14 | Describe any methods used to assess risk of bias due to missing results in a synthesis (arising from reporting biases). | Page 9 |
| **Certainty assessment** | 15 | Describe any methods used to assess certainty (or confidence) in the body of evidence for an outcome. | Page 12 |
| **RESULTS** | | |  |
| **Study selection** | 16a | Describe the results of the search and selection process, from the number of records identified in the search to the number of studies included in the review, ideally using a flow diagram. | Page 11 |
|  | 16b | Cite studies that might appear to meet the inclusion criteria, but which were excluded, and explain why they were excluded. | N/A |
| **Study characteristics** | 17 | Cite each included study and present its characteristics. | Page 9 |
| **Risk of bias in studies** | 18 | Present assessments of risk of bias for each included study. | Page 9-10 |
| **Results of individual studies** | 19 | For all outcomes, present, for each study: (a) summary statistics for each group (where appropriate) and (b) an effect estimates and its precision (e.g. confidence/credible interval), ideally using structured tables or plots. | Page 11-13 |
| **Results of syntheses** | 20a | For each synthesis, briefly summaries the characteristics and risk of bias among contributing studies. | Page 11-13 |
|  | 20b | Present results of all statistical syntheses conducted. If meta-analysis was done, present for each the summary estimate and its precision (e.g. confidence/credible interval) and measures of statistical heterogeneity. If comparing groups, describe the direction of the effect. | Page 11-13 |
|  | 20c | Present results of all investigations of possible causes of heterogeneity among study results. | Page 11-13 |
|  | 20d | Present results of all sensitivity analyses conducted to assess the robustness of the synthesized results. | Page 11-13 |
| **Reporting biases** | 21 | Present assessments of risk of bias due to missing results (arising from reporting biases) for each synthesis assessed. | Page 9-10 |
| **Certainty of evidence** | 22 | Present assessments of certainty (or confidence) in the body of evidence for each outcome assessed. | Page 10 |
| **DISCUSSION** | | |  |
| **Discussion** | 23a | Provide a general interpretation of the results in the context of other evidence. | Page 14-17 |
|  | 23b | Discuss any limitations of the evidence included in the review. | Page 16 |
|  | 23c | Discuss any limitations of the review processes used. | N/A |
|  | 23d | Discuss implications of the results for practice, policy, and future research. | Page 14-17 |
| **OTHER INFORMATION** | | |  |
| **Registration and protocol** | 24a | Provide registration information for the review, including register name and registration number, or state that the review was not registered. | Page 5 |
|  | 24b | Indicate where the review protocol can be accessed, or state that a protocol was not prepared. | N/A |
|  | 24c | Describe and explain any amendments to information provided at registration or in the protocol. | N/A |
| **Support** | 25 | Describe sources of financial or non-financial support for the review, and the role of the funders or sponsors in the review. | N/A |
| **Competing interests** | 26 | Declare any competing interests of review authors. | Page 18 |
| **Availability of data, code and other materials** | 27 | Report which of the following are publicly available and where they can be found template data collection forms; data extracted from included studies; data used for all analyses; analytic code; any other materials used in the review. | N/A |

*From:* Page MJ, McKenzie JE, Bossuyt PM, Boutron I, Hoffmann TC, Mulrow CD, et al. The PRISMA 2020 statement: an updated guideline for reporting systematic reviews. BMJ 2021; 372:n71. doi: 10.1136/bmj.n71

# **PRISMA 2020 Abstract Checklist**

| **Section and Topic** | **Item #** | **Checklist item** | **Reported (Yes/No)** |
| --- | --- | --- | --- |
| **TITLE** | | |  |
| Title | 1 | Identify the report as a systematic review. | Yes |
| **BACKGROUND** | | |  |
| Objectives | 2 | Provide an explicit statement of the main objective(s) or question(s) the review addresses. | Yes |
| **METHODS** | | |  |
| Eligibility criteria | 3 | Specify the inclusion and exclusion criteria for the review. | Yes |
| Information sources | 4 | Specify the information sources (e.g. databases, registers) used to identify studies and the date when each was last searched. | Yes |
| Risk of bias | 5 | Specify the methods used to assess risk of bias in the included studies. | Yes |
| Synthesis of results | 6 | Specify the methods used to present and synthesise results. | Yes |
| **RESULTS** | | |  |
| Included studies | 7 | Give the total number of included studies and participants and summarise relevant characteristics of studies. | Yes |
| Synthesis of results | 8 | Present results for main outcomes, preferably indicating the number of included studies and participants for each. If meta-analysis was done, report the summary estimate and confidence/credible interval. If comparing groups, indicate the direction of the effect (i.e. which group is favoured). | Yes |
| **DISCUSSION** | | |  |
| Limitations of evidence | 9 | Provide a summary of the limitations of the evidence included in the review (e.g. study risk of bias, inconsistency and imprecision). | Yes |
| Interpretation | 10 | Provide a general interpretation of the results and important implications. | Yes |
| **OTHER** | | |  |
| Funding | 11 | Specify the primary source of funding for the review. | N/A |
| Registration | 12 | Provide the register name and registration number. | Yes |

*From:* Page MJ, McKenzie JE, Bossuyt PM, Boutron I, Hoffmann TC, Mulrow CD, et al. The PRISMA 2020 statement: an updated guideline for reporting systematic reviews. BMJ 2021;372:n71. doi: 10.1136/bmj.n71. This work is licensed under CC BY 4.0. To view a copy of this license, visit <https://creativecommons.org/licenses/by/4.0/>
